# EXAMINING LGBT OLDER ADULT CHOSEN FAMILIES USING THE CONVOY MODEL OF SOCIAL RELATIONS

**DOI:** 10.1093/geroni/igae098.1202

**Published:** 2024-12-31

**Authors:** Kelseanne Breder, Walter Bockting

**Affiliations:** New York University, New York, New York, United States; Columbia University / Psychiatric institute, New York, New York, United States

## Abstract

Sexual and gender minority (LGBT) older adults experience heightened levels of social discrimination and minority stress throughout their lives due to their minority identities. Older LGBT adults experienced unique external, systemic barriers to social acceptance, including bans on same-sex marriage, discrimination and negligence during the HIV epidemic, and police brutality during demonstrations such as the Stonewall Riots. LGBT older adults are also more likely to live alone and more likely to be estranged from their families of origin. Taken together, these discriminatory social experiences increase stress exposure and create a demand for an adaptive and protective social network to serve as a counterbalance to minority stress. Many LGBT older adults have developed “chosen families” or social networks composed of non-biological relatives who care for one another as if they are family. The LGBT chosen family is a resilience strategy that has not been examined thoroughly by a theoretical framework. The purpose of this paper is to apply the Convoy Model of Social Relations to examine the LGBT chosen family experience. The convoy metaphor succinctly captures the value of the LGBT chosen family, which functions not only to support members within the chosen family unit, but also to offset exposure to external threats and buffer internalized and externalized homophobia in a heteronormative society. The paper identifies points of convergence and departure between the model and LGBT chosen families and includes an analysis of the role of technology in LGBT older adult social networks. Modifications to the model are proposed.

